# The Diversity, Composition, and Putative Functions of Gill-Associated Bacteria of Bathymodiolin Mussel and Vesicomyid Clam from Haima Cold Seep, South China Sea

**DOI:** 10.3390/microorganisms8111699

**Published:** 2020-10-30

**Authors:** Juan Ling, Hongxiang Guan, Lihua Liu, Jun Tao, Jie Li, Junde Dong, Si Zhang

**Affiliations:** 1 CAS Key Laboratory of Tropical Marine Bio-Resources and Ecology, Guangdong Provincial Key Laboratory of Applied Marine Biology, South China Sea Institute of Oceanology, Chinese Academy of Sciences, Guangzhou 510301, China; lingjuan@scsio.ac.cn (J.L.); lijietaren@scsio.ac.cn (J.L.); dongjd@scsio.ac.cn (J.D.); zhsimd@scsio.ac.cn (S.Z.); 2 Southern Marine Science and Engineering Guangdong Laboratory, Guangzhou 511458, China; 3 Key Laboratory of Gas Hydrate, Guangzhou Institute of Energy Conversion, Chinese Academy of Sciences, Guangzhou 510640, China; liulh@ms.giec.ac.cn; 4 MLR Key Laboratory of Marine Mineral Resources, Guangzhou Marine Geological Survey, Guangzhou 510075, China; 13822116780@139.com

**Keywords:** Haima cold seep, mussel and clam, gill-associated microbial community, potential function

## Abstract

The Haima cold seep, which is one of the two active cold seeps in the South China Sea, is known for its great ecological importance. The seep bivalves are assumed to depend mainly on their bacterial symbiosis for survival and growth. However, information on the bacterial diversity, composition, and putative function of gill-associated of dominant dwelling animals in Haima cold seep remain elusive. Herein, we adopted a high-throughput sequencing of 16S rRNA gene amplicons, and function prediction methods (Functional Annotation of Prokaryotic Taxa (FAPROTAX) and Phylogenetic Investigation of Communities by Reconstruction of Unobserved States (PICURUSTs)) to purposely illustrate the taxonomic and phylogenetic diversity, composition, and putative functions of the symbionts in bathymodiolin mussel *Gigantidas haimaensis* (Bivalvia: Mytilidae: Gigantidas) and vesicomyid clam *Archivesica marissinica* (Bivalvia: Glossoidea: Vesicomyidae). The predominant microbes of both species were Proteobacteria and Gammaproteobacteria on the phylum and class level, respectively. The taxonomic and phylogenetic diversity of gill microbial communities in *G. haimaensis* were significantly different from those in *A. marissinica* (*p* < 0.05). Nine functional groups, including seven carbon-related biogeochemical groups, were identified through the FAPROTAX analysis. However, the most dominant groups for *G. haimaensis* and *A. marissinica* were both chemoheterotrophic. *G. haimaensis* and *A. marissinica* shared many pathways, however, 16 obtained Kyoto Encyclopedia of Genes and Genomes (KEGG) orthologous groups (42.11%) significantly differed between the two species (*p* < 0.05). These findings would provide insight into the functions of microbes in the element cycling and energy flow as well as the host-symbiont relationship of bivalves in the Haima cold seep environment.

## 1. Introduction

Marine cold seeps are characterized by methane-rich fluids, where the material from its sedimentary subsurface back to the seabed and the water column and ultimately some of the methane may even reach the atmosphere [1,2]. Most seeping methane has been demonstrated to be oxidized anaerobically, which are coordinatively performed by the synergistic metabolism of anaerobic methane-oxidizing archaea (ANME) and sulfate-reducing bacteria (SRB) [3]. This process increases the alkalinity of pore waters by producing bicarbonate, leading to precipitation of carbonates [4,5]. The first cold seep was reported in the Florida Escarpment of the Gulf of Mexico in 1984 at a water depth of 3200 m [6]; subsequently, thousands of cold seeps have been discovered globally in oceans [2]. Among which, active cold is characterized by high densities of macrofaunal communities such as mussels, clams, and tubeworm aggregations. Although invertebrates retain the ability of filter-feeding, they mainly gain nutrition through symbioses with chemosynthetic bacteria. Regarding chemosynthetic symbionts, utilization of reduced chemical substances, such as hydrogen sulfide, hydrogen, or methane as electron donors and oxygen as electron acceptors to generate large amounts of energy required for carbon fixation. Therefore, the above underlying mechanism provides a source of carbon to their metazoan host [7].

The metabolism of bacterial symbionts in the gills of the deep-sea mussel Bathymodiolus significantly contributes to their host’s survival through the provision of energy and nutrients [8]. This is vital since the deep-sea mussel Bathymodiolus largely depends on the organic carbon transported from the endosymbionts [9]. Therefore, it is of paramount importance to understand the host-symbiotic relationships in holobiont animals in the cold seep. Moreover, an investigation of gill-associated bacterial community could yield comprehensive knowledge on the adaptation and evolution of these animals to extreme deep-sea habitat [10].

The bacterial symbiosis associated with mussels in deep-sea has extensively been assessed. Sun et al. [11] investigated the gills of *B. platifrons* collected from Formosa cold seep of the South China Sea, and found that the dominant classes of the microbial communities were Gammaproteobacteria and Campylobacterota (previously Epsilonproteobacteria) [12]. Moreover, the mussel bathymodiolus, in most cases live with Gammaproteobacteria, which mainly comprise sulfur-oxidizing chemoautotroph and methane-oxidizer as revealed by fluorescence in situ hybridization (FISH), proteomics, and genomics analysis [13,14,15]. A study by Sun et al. (2017) showed that the existence of metabolic pathways of methanotrophy, assimilatory sulfate reduction, and ammonia in the gill symbiont of *B. platifrons* via metaproteomic analysis, and are among the metabolic processes that enable the host to survive in the extreme deep-sea environment [8]. Moreover, 16S rRNA-encoding gene sequence analyses indicated 93.12% of the obtained OTUs in gills of *Gigantidas haimaensis* were identified as Gammaproteobacteria and the rest were assigned to Campylobacterotal OTUs [12,16]. Some of chemosymbiotic bathymodiolin mussels only host thiotrophic or methanotrophic symbionts, but others may host more diverse chemosyntrophic symbionts depending on mussel species, habitats, and locations [17,18]. Thiotrophic and methanotrophic symbionts has been found to coexist in one individual bathymodiolus mussel, the key metabolic functions of those microbes were significantly different revealed by analyses of their energy and nutrient sources, electron acceptors, and viral defense mechanisms [15]. Furthermore, the vesicomyid clam *Archivesica marissinica* [2] collected from the Haima cold seep was originally described as “*Calyptogena*” *marissinica* C. [19,20]. Its transcriptomes results indicated that the host vesicomyid clam and its symbionts were mutualistic symbioses where the host provides the metabolic needs for its symbiont, whereas the endosymbionts actively supply the nutrients to the hosts.

Microbes play crucial roles in the marine ecosystem by mediating many biogeochemical cycles [21]. The taxonomic microbial community profiles and their function were explored by Louca et al. (2016) using the FAPROTAX database (Functional Annotation of Prokaryotic Taxa) [22]. The results revealed that the sampling environmental conditions greatly impacted the distribution of functional groups in marine microbial communities besides shaping the metabolic niches. The microbial biogeochemical process based on the taxonomic information of the 16S rDNA gene against FAPROTAX database has been investigated in many marine investigations, included the core sediments from the southern Yap Trench [23], Sansha Yongle Blue Hole, Xisha, South China Sea [24] and the inactive hydrothermal vent field, Southwest Indian Ridge [25]. Another tool of phylogenetic investigation of microbial communities by reconstruction of unobserved states (PICRUSt) based on the 16S rRNA gene survey was widely adopted in the microbial community studies [26,27]. The PICRUSt results could provide the information Kyoto encyclopedia of genes and genomes (KEGG) ortholog (KOs) predictions for the gene family of the investigated sample, and this information could be further summarized as pathway-level categories [28]. These categories include carbohydrate and lipid metabolism, energy metabolism, environmental information processing, and so on [26].

The active cold seeps Site F (Formosa Ridge) and Haima seep were discovered in the South China Sea in 2013 and 2015, respectively. High densities of megafaunal communities such as mollusks, polychaete worm, crustacean, and glass sponge were ubiquitously observed at both seep sites [2,29]. The Haima cold seep, located in the Qiongdongnan Basin, is still in an early stage of community succession [2,30,31]. Both mussel *G. haimaensis* and vesicomyid clam A. marissinica were identified as new species [16,19]. Based on the morphological and genetic characteristics analyses, *G. platifrons*, *G.childressi*, and *G. mauritanicus* were demonstrated to be the three most closely related species with *G. haimaensis*. Moreover, *A. marissinica* is indicated to be a member of the family vesicomyidae and is a sister of *Calyptogena similaris* [20].

However, information on the gill-associated bacterial communities of bathymodiolin mussel and vesicomyid clam in Haima cold seep is limited. Therefore, 16S rRNA high-throughput sequencing in combination with two functional prediction methods (FAPROTAX and PICURUSTs) was employed in this study to illustrate the bacterial diversity, composition, and putative function of the two types of bivalves. The results would enrich the knowledge on the associated bacterial community structure and functions of the dominant dwelling animals in cold seep habit as well as facilitating further studies on the host-symbionts relationship.

## 2. Materials and Methods

### 2.1. Sample Collection

**Figure 1:**
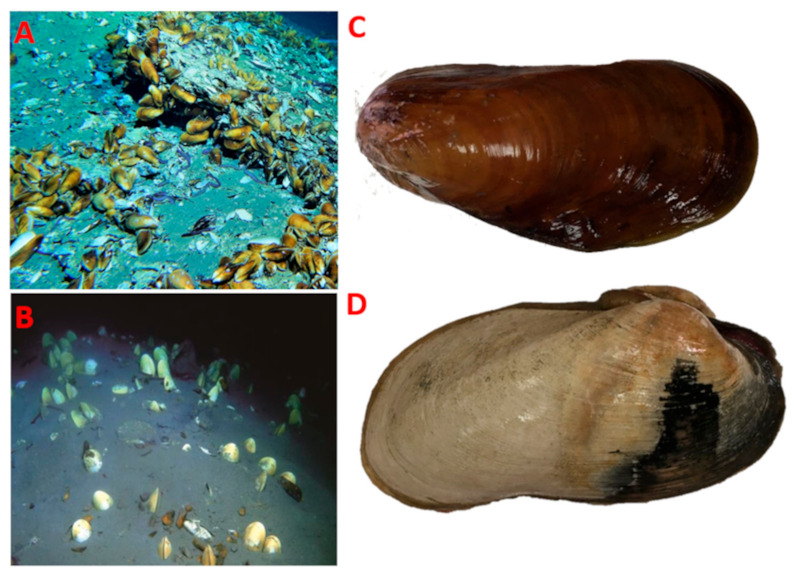
Photograph of the two species of bivalves *Gigantidas haimaensis* and *Archivesica marissinica* from Haima cold seep. The in situ photo showing the population of *G. haimaensis* (**A**) and *A. marissinica* (**B**). The photo of single *G. haimaensis* (**C**) and *A. marissinica* (**D**).

The bathymodiolin mussel *G. haimaensis* (A) and vesicomyid clam *A. marissinica* (B) were sampled from Haima cold seep area (16.73° N, 110.475° E) in the northwest slope of the South China Sea on May 2019 (Figure 1) [32]. The species of *G. haimaensis* were sampled during the dive 2019-HM-ROV01 at water depth of 1380 m, whereas the *A. marissinica* species were sampled at approximately water depth of 1390 m during the dive 2019-HM-ROV05 using the ROV Haima on-board the research vessel (R/V) Haiyang 6, Guangzhou Marine Geological Survey (China). All the investigated samples were analyzed in triplicate. The samples were frozen immediately at −80 °C after collection, and at the end of the cruise, all samples on dry ice were transported to the lab. The samples of the bathymodiolin mussel and vesicomyid clams were dissected into gill and other tissues, then immediately stored at −80 °C for further molecular analysis. These two species have been identified on morphological characteristics and molecular methods (mitochondrial genes cytochrome oxidase I) in the former investigations [16,20]. *G. haimaensis* in this study is the same bathymodiolin mussel species as the study of Xu et al. (2019) [16], and *A.marissinica* is the same vesicomyid clam species as the investigation by Chen et al. (2018) [20].

### 2.2. DNA Extraction, PCR Amplification, and 16S rDNA Amplicon Sequencing

The community DNA of the gill-associated microbes extracted from approximately 1 g of each sample (wet weight) using the CTAB method according to Stewart and Via [33]. The concentration and quality (assessed by OD 260 /OD 280 ratios) of the extracted DNA were assessed by NanoDrop ND-2000 spectrophotometer (Thermo Fisher Scientific, Wilmington, DE, USA), following their integrity determination on 1% agarose gel electrophoresis. The universal primer pair 515F (5′-GTG YCA GCM GCC GCG GTA-3′) and 907R (5′-CCG YCA ATT YMT TTR AGT TT-3′) were employed to amplify the V4-V5 region of the 16s rRNA gene of the gill-associated microbial community. The PCR mixture (25 µL) contained 2.5 µL of the AmpliTaq™ Gold PCR Master Mix (2×) (Applied Bio-systems, Foster, CA, USA), 0.4 µM of each primer, and about 20 ng DNA template. The following PCR conditions were used: at 95 °C for 5 min, followed by 25 cycles of 95 °C for 30 s, 55 °C for 30 s, 72 °C for 90 s, and a final extension at 72 °C for 10 min. Subsequently, the PCR products were purified and quantified, and the qualified PCR products were sequenced on the Illumina MiSeq platform (Illumina Inc., San Diego, CA, USA) with a paired-end sequencing strategy (2 × 300 bp). The raw sequence data sequencing data were deposited to GSA (https://bigd.big.ac.cn/gsa/) with Bioproject number PRJCA002306.

### 2.3. Sequence Analysis

The resultant amplicon sequencing bioinformatics were performed with EasyAmplicon v1.0 [34]. The paired-end sequence data were merged, quality filtered and dereplicated using VSEARCH v2.15 subcommand --fastq_mergepairs, --fastx_filter, and --derep_fulllength, respectively [35]. Thereafter, the left sequences were denoised and assembled using the unoise3 pipeline in the USEARCH at a 100% identify threshold to obtain the exact sequence variants (ESVs) denoted as ZOTUs (denoised into zero-radius operational taxonomic units) according to Edgar (2018) [36]. The RDP classifier (https://rdp.cme.msu.edu/classifier/) could provide the taxonomic assignments to ZOTUs. Moreover, the chloroplast sequences were discarded during further analysis. The neighbor-joining phylogenetic trees of top 10 ZOTUs of *G. haimaensis* and *A. marissinica* and their reference sequences in NCBI were constructed using MEGA software (v7.0.18), respectively [37]. The reference sequences were selected by comparison with the GenBank database using BLAST (https://blast.ncbi.nlm.nih.gov/Blast.cgi). Sequence of selected ZOTUs’ and their references were firstly aligned by the ClustalW method using MEGA. Then, the aligned sequences were employed to construct the neighbor-joining phylogenetic tree with 1000 bootstrap replicates with MEGA. For the circular phylograms construction, all the targeted ZOTUs in this study were firstly aligned by the ClustalW method in MEGA, and further visualized by Interactive Tree of Life (iTOL) (http://itol.embl.de/).

### 2.4. Statistical Analysis

The R software (R Core Team, 2018) was used for the majority of analyses in this study with the package of “Vegan” and “Amplicon” [38,39]. The alpha diversity index (ESV Richness, Simpson, Shannon, and Faith’s phylogenetic diversity) was statistically analyzed after rarefaction of the obtained ZOTU table, with all data sets of samples normalized to 34,128 reads (the minimum number of effective sequences for the six samples) per subsample. Good’s coverage estimators representing the subsample coverage were also calculated using the Vegan package. Variations between different species (beta diversity) were determined using principal component analysis (PCA) ordination based on rarefied OTU abundances. The permutational multivariate analysis of variance (PERMANOVA), multiple regression analysis (MRPP), and analysis of similarity (ANOSIM) implemented in the R package vegan was used to test the significance of the differences between two species of microbial communities [25]. The obtained rarefied ZOTUs table and KEGG pathways profiles were further subjected to the Statistical Analysis of Metagenomic Profiles (STAMP) software package [40].

### 2.5. FAPROTAX Analysis

The FAPROTAX database with the normalized ZOTU table [22] can predict the biogeochemical processes of the gill-associated microbes. Accordingly, the FAPROTAX database mainly focuses on marine and aquatic biogeochemistry. Hence, the database and its software are available at http://www.zoology.ubc.ca/louca/FAPROTAX. This database is a manually developed from literature on cultured prokaryotes at the genus level. In total, it comprises 90 functional groups having the main processes of carbon, nitrogen, and sulfur cycling.

### 2.6. PICRUSt Analysis

This analysis relies on the obtained table of operational taxonomic units (OTUs) for each sample against the Greengenes ribosomal RNA database (release of August 2013) at 97% similarity [41,42]. The obtained OTUs were normalized by dividing their abundances by known or predicted 16S rDNA gene copy number abundances before their final predictions, and the resultant KOs was used to express the functional information of the microbial community. The predicted functional counts were rarefied to the same depth for further analysis. KEGG orthologous gene data refers to the equivalent genes and gene products (e.g., RNA and proteins) that were produced in different organisms. Moreover, they contained subgroups of cellular processes, environmental information processing, genetic information processing, metabolism, organismal systems, and human diseases (Level 1). The accuracy of function predictions depends on how closely the obtained microbes in a given sample are related to the microbes with sequenced genome representatives, as measured by the Nearest Sequenced Taxon Index (NSTI) where the lower values indicate a closer mean relationship [26]. In addition, KEGG Orthology terms of the microbial communities predicated by PICRUSt were further analyzed by STAMP [40].

## 3. Results

### 3.1. The Taxonomic and Phylogenetic Composition of the Gill-Associated Microbial Communities

**Figure 2:**
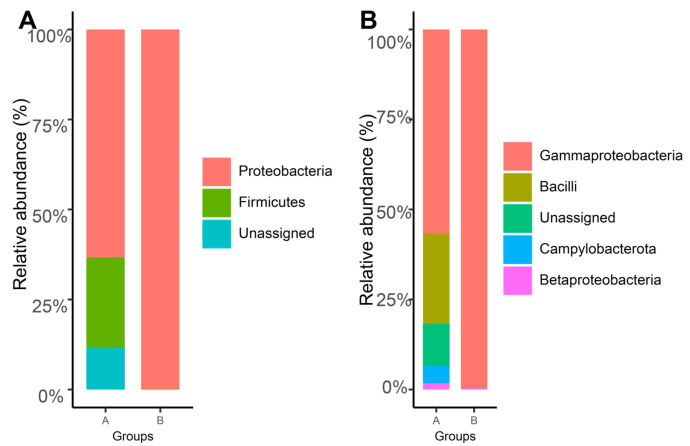
The taxonomic composition of bathymodiolin mussel *G. haimaensis* (Group A) and vesicomyid clam *A. marissinica* (Group B) at the phylum level (**A**) and the genus level (**B**), respectively.

**Figure 3:**
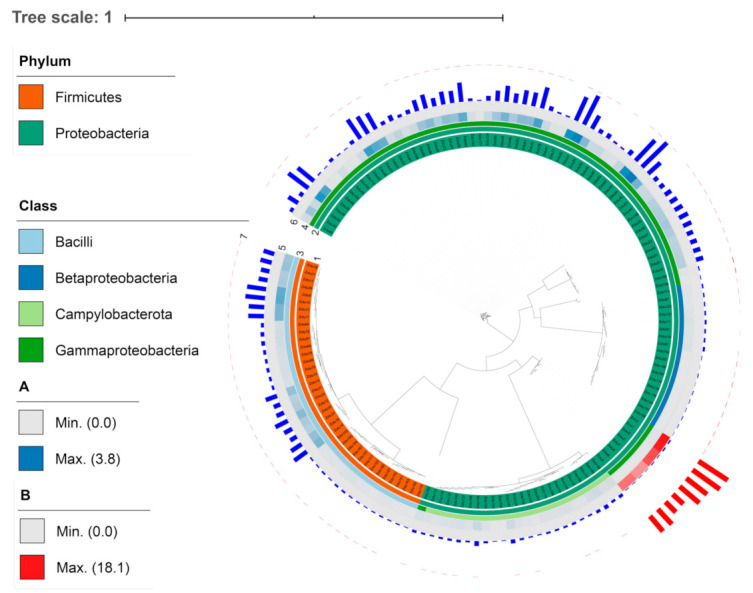
Phylogenetic relationship and annotation of ZOTUs (zero-radius operational taxonomic units) obtained in the gill of bathymodiolin mussel *G. haimaensis* and vesicomyid clam *A. marissinica*. From inner to outer: ring 1 represents all the ZOTUs detected this study; rings 2 and 3 indicate the phylogenetic classification of ZOTUss at the phylum and class level, respectively, and their legends were in the left part; rings 4 and 5 with heatmaps show the relative abundance of ZOTUs in abundance in each group. The legends were in the left part with *G. haimaensis* (Group A) and *A. marissinica* (Group B). The outer rings 6 and 7 with histograms demonstrated a relative abundance of ZOTUs in *G. haimaensis* (blue) and *A. marissinica* (in red).

A total of 201 ZOTUs were identified from the samples. Notably, the six ZOTUs of the non-bacterial sequence (mostly chloroplasts) were not included for further analysis. The sequencing depth was 34,128 reads for all samples after resampling. The sequencing effort of *G. haimaensis* (A) and *A. marissinica* (B) was shown to reach the saturation stage with increasing numbers of samples, and hence, this investigation has captured most of the gill-associated microbes (Figure S1). Moreover, the taxonomic composition of the microbial communities at the phylum (Figure 2A) and the class (Figure 2B) level were illustrated differently, respectively. Accordingly, the ZOTUs were mainly assigned to the two phyla namely Proteobacteria (57.44%) and Firmicutes (19.49%). At the phylum level, the ZOTUs affiliated to phylum Proteobacteria could be subgrouped into classes Betaproteobacteria, Gammaproteobacteria, and Campylobacterota, where the dominant class was Gammaproteobacteria (35.38%). The taxonomic composition of *A. marissinica* only consisted of phylum Proteobacteria at the phylum level and consisted of classes Gammaproteobacteria and Betaprotebacteria, where *G. haimaensis* had four classes (Figure 2B). From the circular phylograms (Figure 3), it showed the taxonomical composition and relative abundance of *G. haimaensis* (Group A) and *A. marissinica* (Group B).

**Figure 4:**
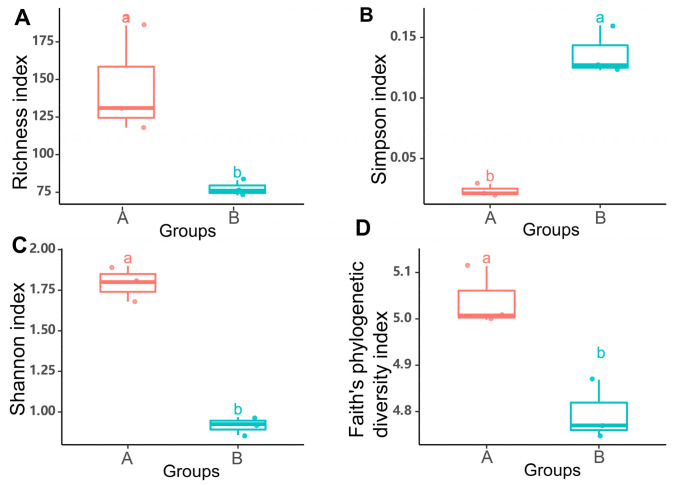
The alpha diversity indices including (**A**) ZOTUs (zero-radius operational taxonomic units) richness, (**B**) Simpson, (**C**) Shannon diversity index, and (**D**) Faith’s phylogenetic diversity index for obtained gill-associated microbial communities of *G. haimaensis* (Group A) and *A. marissinica* (Group B).

Illustrations on the taxonomic (Richness, Simpson, and Shannon diversity index) and Faith’s phylogenetic alpha diversity index of the microbial communities are indicated in Figure 4. Based on these indices, significant differences between *G. haimaensis* (Group A) and *A. marissinica* (Group B) were noted (*p* < 0.05). Moreover, higher Richness index, Shannon index, and Faith’s phylogenetic diversity index existed in *G. haimaensis*. For *G. haimaensis*, the average richness index was 145, while that of *A. marissinica* was 77. At the phylogenetic level, the average Faith’s phylogenetic diversity index of *G. haimaensis* was 5.03, and the value was 4.80 for *A. marissinica* (*p* < 0.01).

**Figure 5:**
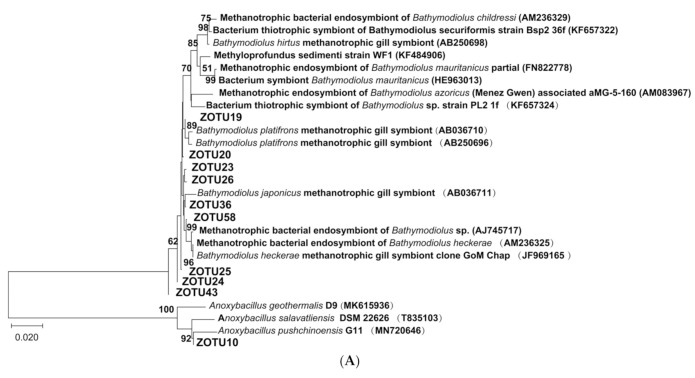

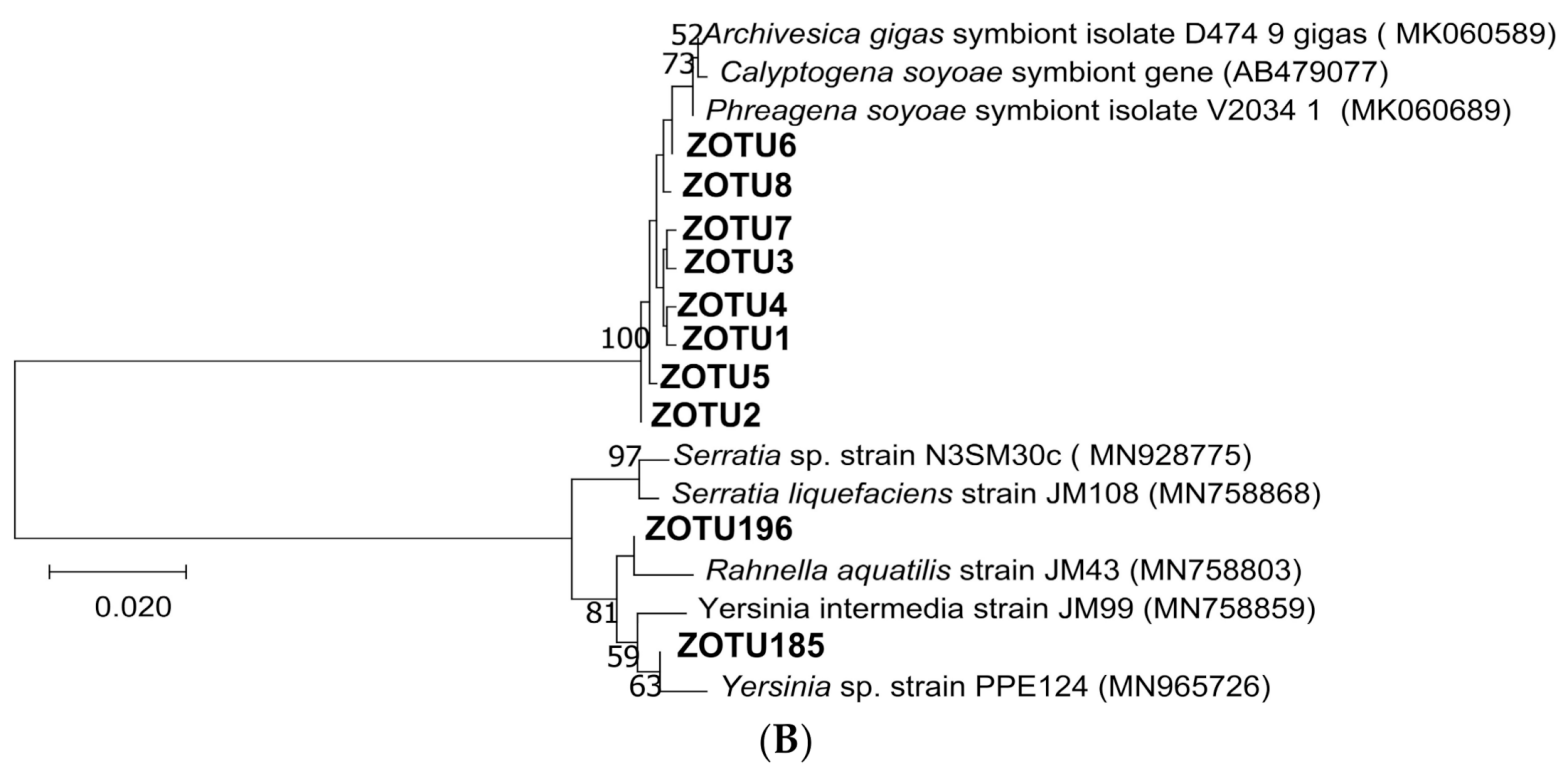
Phylogenic tree of the gill-associated microbes in *G. haimaensis* (**A**) and *A. marissinica* (**B**) for their top 10 high abundance ZOTUs (zero-radius operational taxonomic units) and their reference sequences, respectively, inferred from the Neighbor-Joining (NJ) analysis. The ZOTUs in (**A**) indicated the top 10 ZOTUs in *G. haimaensis*, and the ZOTUs in (**B**) indicated the top 10 ZOTUs in *A. marissinica*. The bootstrap values (> 50%) of relevant nodes are shown based on 1000 replicates. Sequences from this study are shown in bold. Scale bars: 2% of the estimated sequence divergence.

The phylogenetic taxonomy of the top 10 ZOTUs and their relative abundance in bathymodiolin mussel *G. haimaensis* and vesicomyid clam *A. marissinica,* respectively, were shown in Table 1. Nine of the top 10 ZOTUs in *G. haimaensis* belonged to class Gammaproteobacteria, and their relative was 25.45%. Only ZOTU10 belonged to phylum Bacilli, and its relative abundance was 2.17%. For *A. marissinica,* all the top 10 ZOTUs belonged to class Gammaproteobacteria, and their relative abundance was 98.86%. The phylogenetic trees were constructed for *G. haimaensis* and *A. marissinica* based on their top 10 ZOTUs, respectively (Figure 5). As illustrated in Figure 5A, the top 10 ZOTUs of *A. marissinica* could form two well-supported clades. One clade comprised of nine ZOTUs, and they clustered closely with previously investigated methanotrophic gill symbionts of bathymodioline mussels. Only ZOTU 10 was in the other clade, and closely clustered closely with microbes from the genus Anoxybacillus. For *A. marissinica,* the 10 ZOTUs formed two clades with ZOTUs 1–8 formed one clade, and ZOTU196 and ZOTU185 consisted of another clade. The ZOTU 196 was phylogenetically close to *Rahnella aquatilis* JM 43, while ZOTU 185 had high similarity with *Yersinina* sp. PPE124 (Figure 5B).

### 3.2. Variations of the Microbial Communities in G. haimaensis and A. marissinica

**Figure 6:**
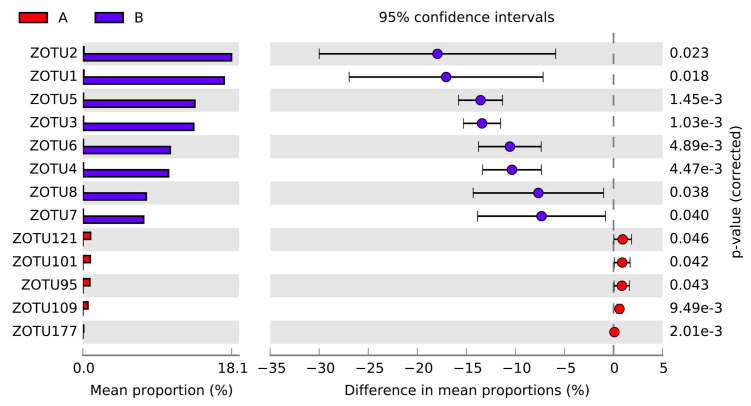
Variation analysis of gill-associated microbial community between *G. haimaensis* (Group A) and *A. marissinica* (Group B) at the ZOTUs (zero-radius operational taxonomic units) level. Red bars and dots stand for *G. haimaensis*, and blue bars and dots stand for *A. marissinica*.

Results from the principal component analysis were visualized based on the first two principal axes in Figure S2, where the gill-associated microbes of *G. haimaensis* were shown to be distinct from that of A. marissinica based on the axis 1. Although beta diversity analysis did not show significant differences between the microbial communities of *G. haimaensis* (A) and *A. marissinica* (B) analyzed by MRPP, PERMANOVA, and Anosim (*p* > 0.05) at the community level. However, 13 OTUs were significantly different between the two species at the ZOTU level analyzed by STAMP (Figure 6). Most of the ZOTUs belonged to Gammaproteobacteria (69.23%, namely, ZOTU1, ZOTU2, ZOTU3, ZOTU4, ZOTU5, ZOTU6, ZOTU7, ZOTU8, and ZOTU177) where the rest were unassigned. The highest relative mean abundance ZOTU in *A. marissinica* was ZOTU2, and the mean relative abundance was 18.07%, while for that of *G. haimaensis*, the value was 0.16%.

### 3.3. Biogeochemical Process Profiling Predicted by FAPROTAX Database

**Figure 7:**
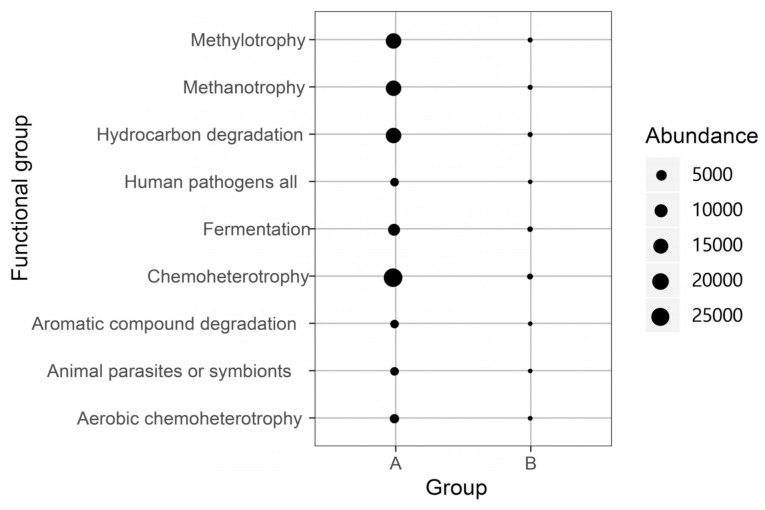
The bubble plot of the function groups predicted against the FAPROTAX database for *G. haimaensis* (Group A) and *A. marissinica* (Group B).

FAPROTAX has been widely used for the microbial functional annotation of the biogeochemical process. Exactly 51.85% of ZOTUs were assigned to at least one group in this study, following the identification of nine functional groups for *G. haimaensis* and *A. marissinica* after mapping to the FAPROTAX database (Figure 7). Although the nine functional groups could be detected in Group A and B, some of the functional groups of their relative abundance were significantly different (*p* < 0.05). For instance, the most dominant function group for *G. haimaensis* was in the chemoheterotrophy group, with the mapped ZOTUs accounted for 78.76% of all ZOTUs. Methanotrophy, methylotrophy, and hydrocarbon degradation were found to be dominant function groups for G. haimaensis. Among these three groups, their ZOTUs represented 50% of all ZOTUs. However, for *A. marissinica*, the relative abundance of the predicted functional groups was very low, while the highest relative abundance of functional group was chemoheterotrophy (0.89%).

### 3.4. Potential Functional Profiling Analyzed by PICRUSt Analysis

**Figure 8:**
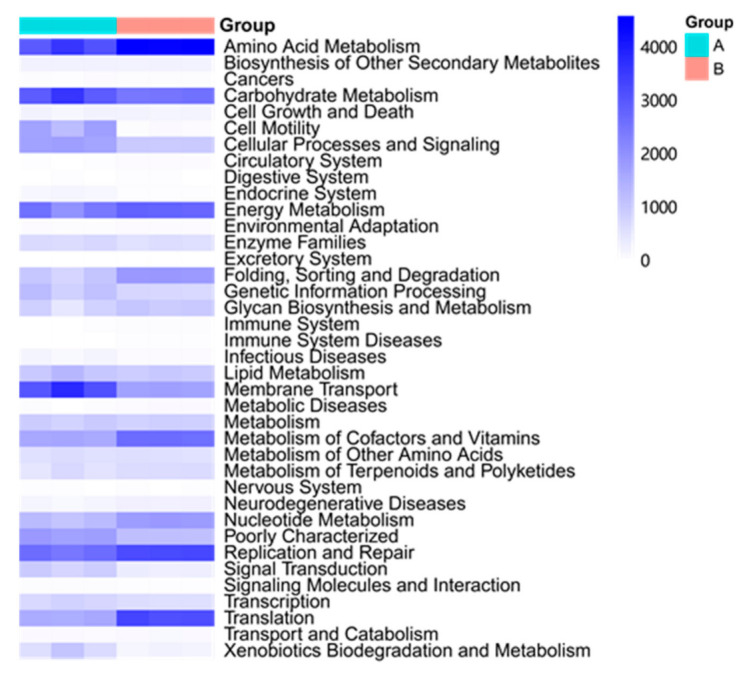
The heatmap of KO groups predicted by PICRUSt analysis in *G. haimaensis* (Group A) and *A. marissinica* (Group B) at KEGG level 2.

**Figure 9:**
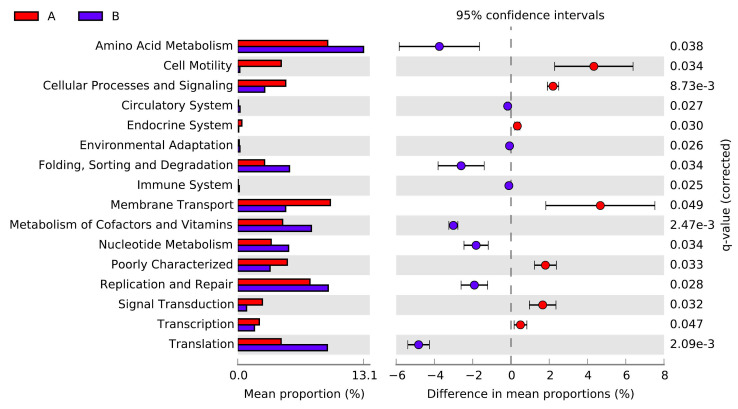
Variation analysis of gill-associated microbial community between *G. haimaensis* (Group A) and *A. marissinica* (Group B) at KEGG level 2. Red bars and dots stand for *G. haimaensis*, and blue bars and dots stand for *A. marissinica*.

The PICRUSt analysis yielded that 38 of 43 were level 2 KOs in the microbial communities of *G. haimaensis* and *A. marissinica* (Figure 8). Notably, two species shared some KOs despite having significant differences in some of their KEGG pathways. Moreover, these two species demonstrated their potential functions in the pathways of Amino Acid Metabolism, Carbohydrate Metabolism, Energy metabolism, and the others. Based on the STAMP findings, it was evident that the two species showed significant variations in 16 KOs (42.11%) (*p* < 0.05) (Figure 9). For instance, *A. marissinica* had higher potential functional activity in the aspect of Amino Acid Metabolism, Replication and Repair, and Translation while the activity of Membrane transport and Celluar Process and signaling Transduction were higher in *G. haimaensis*.

## 4. Discussion

Studies on microbial communities, especially the compositional and functional diversities of chemosynthetic microbial communities, have attracted great attention in the extremophilic deep-sea biotopes. The advancement of new research tools has immensely accelerated research progress [11,43,44]. In this study, the obtained ZOTUs were assigned to two phyla and four classes. The phylum Proteobacteria and class Gammaproteobacteria were predominant on the phylum and class level, respectively. The taxonomy composition of bathymodiolin mussel *G. haimaensis* and vesicomyid clam *A. marissinica* were shown to be distinct (Figure 2). Moreover, the alpha diversity in *G. haimaensis* was significantly higher than in *A. marissinica* both at the taxonomic and phylogenetic levels (Figure 4). Besides the phylum Proteobacteria, the phylum Firmicutes has also been found in *G. haimaensis* in our study. Microbes affiliated to this phylum were widely distributed in diverse ecological habitats with the spore-forming ability to cope with environmental challenges, such as heat, desiccation, presence of organic solvents and oxidizing agents, and UV irradiation [45]. The gill-associated microbes of bathymodiolin mussel *G. haimaensis* collected in 2018 in Haima cold seep mainly consisted of Gammaproteobacteria (93.12%) and Campylobacterota (6.88%) at the class level, whereas the most predominant Gammaproteobacterium was methanotrophic microbe predicted by phylogenetic analysis [16]. Similarly, our investigation result showed that nine of the top 10 high abundant ZOTUs in *G. haimaensis* clustered closely with the methanotrophic microbe, and they belonged to class Gammaproteobacteria (Figure 5A). Moreover, Coykendall et al. (2019) has reported that a methanotrophic phylotype were predominate in all gill microbial samples from Baltimore Canyon seep located in the U.S. Atlantic margin [46]. As for *A. marissinica* in Haima cold seep, no study was conducted on its taxonomic composition of microbial communities available, although some research has been conducted on the host-symbiont interactions [9].

The host individuals’ genetic diversity would influence the relationships between hosts and their symbionts [15]. Although bathymodiolin mussels and vesicomyid clams were both dominant and widespread in the deep sea, they obtain microbes in different ways. Mussels could take in free-living bacteria from the surrounding environment, while vesicomyid clams mainly inherit maternal transmission [19]. With their different adaption process, they formed their unique mode of mutualistic relationship with their symbionts by horizontal or vertical transmission to sustain the primary physical metabolism [19]. All these modes may affect their living styles. As observed in the previous investigation, bathymodiolus mussels obtained their nutrients by utilizing the lysosomal enzymes to digest the symbiont [14]. Simultaneously, the vesicomyid clams were shown to take up water and hydrogen sulfide in the sediment through their foot [47].

Furthermore, Ohishi et al. (2016) reported that the endosymbionts of the deep-sea clam *Calyptogena okutanii* were thioautotrophic bacteria and can rely on hydrogen sulfide as the primary energy [47]. However, the result of the transcriptome analysis of different organs in *A. marissinica* revealed that sulfide oxidation pathways were essential for the metabolism of the endosymbionts [19]. Moreover, a comparative transcriptomic analysis was conducted between deep-sea bathymodiolin mussels and shallow-water mussels [48], and their results suggested that they all possessed the ability to adapt to different harsh habits with different genes of the same gene family highly expressed for a different source of mussels [48]. Moreover, some vital metabolic activities, such as lysosome activity, apoptosis, and immune reactions, were significant in preserving the stability for the mutually beneficial symbiosis [48].

Microbes play diverse roles in the deep-sea symbionts. Previous studies demonstrated that two types of bacterial symbionts species, including sulfur-oxidizing (SOX) and methane-oxidizing (MOX) symbiont, were widespread and could coexist in the deep-sea bathymodiolus mussels [16]. Since they both resided in the specialized gill epithelial cells called bacteriocytes, they could utilize the reduced compounds from hydrothermal fluids as energy sources for carbon fixation to support their host’s growth and reproduction [16]. MOX microbes may participate in methanotrophy process. In our study, we discovered the methanotrophy-related microbes in the *G. haimaensis* accounting for 48.01% in their communities. The FAPROTAX result showed that other biogeochemical processes influenced by gill-associated microbes included chemoheterotrophy, methylotrophy, hydrocarbon degradation, aerobic chemoheterotrophy, aromatic compound degradation, and fermentation. Methanotrophs were dominant in the gills of *B. platifrons* in the cold seep, South China Sea [9], which was in agreement with our result that the abundance of the predicted methanotrophic group of *G. haimaensis* accounted for about 50% of the total microbes obtained (Figure 7).

The KEGG result showed that the investigated two species both demonstrated high relative abundance in amino acid metabolism, carbohydrate metabolism, and energy metabolism (Figure 8). Hence, these results attribute that the aforementioned three functions could meet the basic growth needed and should be highly expressed even in the oligotrophic deep-sea area. Many unassigned microbes had been found in the *A. marissinica*, and their functions in the biogeochemical process of the ocean remains unknown. Therefore, their taxonomic affiliation, metabolism, and genetic potential necessitate further studies in the future.

## 5. Conclusions

Herein, we investigated the gill-associated microbes of bathymodiolin mussel *G. haimaensis* and vesicomyid clam *A. marissinica* collected from Haima cold seep, South China Sea. Notably, from our findings, we demonstrated significant differences in the alpha taxonomic and phylogenetic diversity, composition, and putative functions between the two bivalve species (*p* < 0.05). However, for beta diversity, these two species demonstrated no significant differences at the microbial community level (*p* > 0.05). Moreover, 13 ZOTUs were significantly different distributed in mussel *G. haimaensis* and vesicomyid clam A. marissinica at the ZOTU level. Nine of the top 10 ZOTUs in *G. haimaensis* were phylogenetically related to methanotrophic gill symbionts of other bathymodioline mussels. The dominant taxonomy among them included both Proteobacteria and Gammaproteobacteria at the phylum and class level, respectively. Results of putative function prediction methods, including FAPROTAX and PICURUST, revealed that although there were many functions shared by these two species, many were significantly different. These results would provide an insight into the gill-associated microbes’ biogeochemical function and the host-symbionts relationship in the cold seep environment. To better understand the host–symbiont relationship, metatranscriptomic and metaproteomic approaches will be adopted to in-depth investigate the microbial populations in these extremophilic microbial communities in the future.

## Figures and Tables

**Table 1:** Phylogenetic classification and their relative abundance of the top 10 ZOTUs (zero-radius operational taxonomic units) in bathymodiolin mussel *Gigantidas haimaensis* and vesicomyid clam *Archivesica marissinica*.

ZOTU ID	The Relative Abundance		Phylum	Class
	*G. haimaensis*	*A. marissinica*		
**TOP 10 ZOTUs in *G. haimaensis***				
ZOTU24	3.83%	0.00%	Proteobacteria	Gammaproteobacteria
ZOTU25	3.52%	0.01%	Proteobacteria	Gammaproteobacteria
ZOTU19	3.10%	0.02%	Proteobacteria	Gammaproteobacteria
ZOTU20	2.83%	0.02%	Proteobacteria	Gammaproteobacteria
ZOTU26	2.74%	0.01%	Proteobacteria	Gammaproteobacteria
ZOTU36	2.53%	0.02%	Proteobacteria	Gammaproteobacteria
ZOTU43	2.38%	0.02%	Proteobacteria	Gammaproteobacteria
ZOTU23	2.30%	0.02%	Proteobacteria	Gammaproteobacteria
ZOTU58	2.22%	0.01%	Proteobacteria	Gammaproteobacteria
ZOTU10	2.17%	0.01%	Firmicutes	Bacilli
**TOP 10 ZOTUs in *A. marissinica***				
ZOTU2	0.16%	18.07%	Proteobacteria	Gammaproteobacteria
ZOTU1	0.15%	17.38%	Proteobacteria	Gammaproteobacteria
ZOTU5	0.08%	13.64%	Proteobacteria	Gammaproteobacteria
ZOTU3	0.10%	13.31%	Proteobacteria	Gammaproteobacteria
ZOTU6	0.07%	10.66%	Proteobacteria	Gammaproteobacteria
ZOTU4	0.08%	10.39%	Proteobacteria	Gammaproteobacteria
ZOTU8	0.06%	7.61%	Proteobacteria	Gammaproteobacteria
ZOTU7	0.05%	7.44%	Proteobacteria	Gammaproteobacteria
ZOTU185	0.13%	0.20%	Proteobacteria	Gammaproteobacteria
ZOTU196	0.08%	0.16%	Proteobacteria	Gammaproteobacteria

## Supplementary Materials

The following are available online at https://www.mdpi.com/2076-2607/8/11/1699/s1, Figure S1: Rarefaction curves of detected bacterial species of the gill-associated microbes of bathymodiolin mussel *G. haimaensis* (A) and vesicomyid clam *A. marissinica* (B), Figure S2: The principal component analysis of the gill-associated microbes of bathymodiolin mussel *G. haimaensis* (A) and vesicomyid clam *A. marissinica* (B).
