# Supplementary material for: The Diversity, Composition, and Putative Functions of Gill-Associated Bacteria of Bathymodiolin Mussel and Vesicomyid Clam from Haima Cold Seep, South China Sea
Source: Microorganisms. 2020 Oct 30;8(11):1699. doi: 10.3390/microorganisms8111699 (PMC7694083; doi:10.3390/microorganisms8111699)
Supplement: Supplementary file 1 [file microorganisms-08-01699-s001.pdf]

microorganisms

Supplementary Figures:

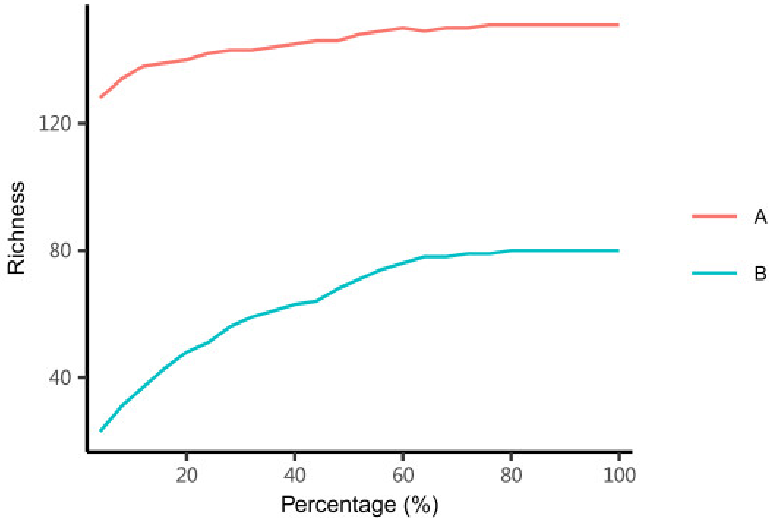

Figure S1. Rarefaction curves of detected bacterial species of the gill-associated microbes of *Gigantidas haimaensis* (A) and *Archivesica marissinica* (B).

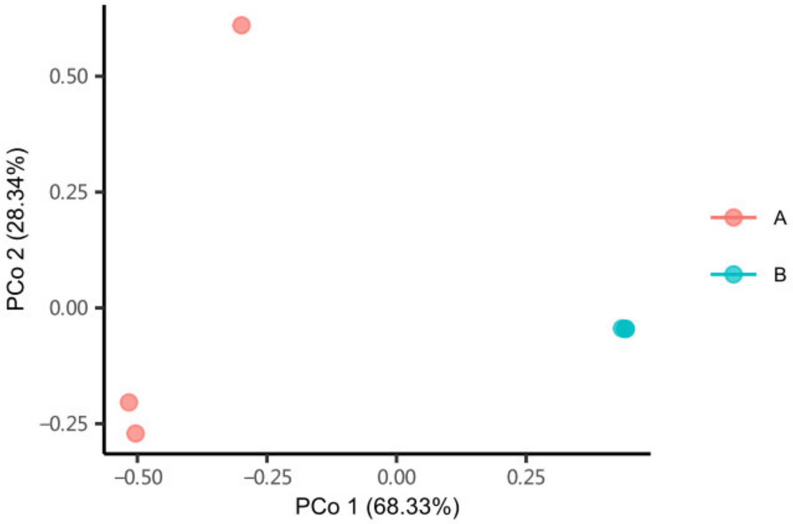

Figure S2. The principal component analysis of the gill-associated microbes of *G. haimaensis* (A) and *A. marissinica* (B)

© 2020 by the authors. Licensee MDPI, Basel, Switzerland. This article is an open access article distributed under the terms and conditions of the Creative Commons Attribution (CC BY) license (http://creativecommons.org/licenses/by/4.0/).
